# Renal Sinus Angiomyolipoma: An Unusual Cause of a Pelvicalyceal Mass

**DOI:** 10.7759/cureus.90330

**Published:** 2025-08-17

**Authors:** Muhammad Ayub, Sobia Ahmed, Quang Dai La, Eashwar Krishna, Aiman Baloch, Shazia Shabbir, Sumayya Bashir, Farzana Rahim, Francis Pryor

**Affiliations:** 1 Radiology, Bolan Medical Complex Hospital, Quetta, PAK; 2 Biology, Texas A&M University, College Station, USA; 3 Medicine, The Innovative STEMagazine 501(c)3, College Station, USA; 4 Molecular and Cell Biology, University of Connecticut, Storrs, USA; 5 Medicine, Mekran Medical College Turbat, Balochistan, PAK; 6 Medicine, Lake Erie College of Osteopathic Medicine, Erie, USA

**Keywords:** angiomyolipoma, ct imaging, flank pain, hematuria, histopathology, nephron-sparing surgery, pelvicalyceal system, renal mass, renal sinus, ureteropelvic junction obstruction

## Abstract

Renal angiomyolipomas (AMLs) are typically benign tumors of the renal cortex, often discovered incidentally. Although rare, AMLs can originate in the renal sinus and extend into the pelvicalyceal system, leading to obstructive uropathy and mimicking urothelial malignancies. We present the case of a 23-year-old female who was referred to our institution with right flank pain and hematuria. Imaging revealed a fat-containing mass in the right renal sinus extending into the pelvicalyceal system and causing ureteropelvic junction obstruction. A presumptive diagnosis of AML was made based on CT characteristics, which was later confirmed histopathologically following surgical excision. This case emphasizes the importance of recognizing the characteristic imaging features of AMLs, particularly in unusual locations, and highlights the role of biopsy in guiding conservative, nephron-sparing management.

## Introduction

Angiomyolipoma (AML) is the most prevalent benign neoplasm of the kidney, composed of blood vessels, smooth muscle, and fat, and is classified within the perivascular epithelioid cell tumor (PEComa) family [1,2]. AMLs typically arise in the renal cortex, but there are uncommon reports of renal sinus lesions that extend into the collecting system, sometimes mimicking urothelial carcinoma [1,2].

About 80-90% of AMLs are sporadic, most often occurring in middle-aged females, while the remaining ~20% are associated with syndromic conditions such as tuberous sclerosis complex (TSC) or pulmonary lymphangioleiomyomatosis, and less commonly with neurofibromatosis type 1 or Von Hippel-Lindau disease [3-5]. Renal AMLs associated with these syndromes are typically multiple, bilateral, larger, diagnosed at younger ages, and carry a greater risk of rapid growth and bleeding compared with sporadic AMLs [3,4,6].

Approximately 0.3-3% of all renal neoplasms have been identified as AMLs, with their prevalence in the general population estimated at 0.13-2.2%. About 20% of AMLs are associated with TSC, usually presenting bilaterally at a younger age [1]. Pathologically, AMLs are divided into classic (triphasic) AMLs, which contain adipose, vascular, and smooth muscle elements, and epithelioid AMLs, which may demonstrate aggressive behavior and even malignant potential [1].

Presenting symptoms may include flank pain, hematuria, and, less commonly, spontaneous hemorrhage, particularly in lesions >4 cm [7]. Some AMLs, especially fat-poor or fat-invisible variants, pose diagnostic challenges, as they may mimic renal cell carcinoma (RCC) radiologically, particularly on ultrasound and CT [8].

CT remains the cornerstone of imaging, with classic AMLs demonstrating macroscopic fat that can be readily identified. However, fat-poor variants are more challenging, particularly when arising in atypical locations such as the renal pelvis, and may cause diagnostic difficulty [1]. Fat-poor AMLs may appear hyperattenuating and be misidentified on unenhanced CT as RCC, requiring MRI or biopsy for accurate differentiation, especially when occurring in unusual sites such as the renal pelvis [8].

We report the case of a 23-year-old female who presented with right flank pain and hematuria. Non-contrast CT revealed features consistent with a renal sinus AML extending into the collecting system. Histopathology following excisional biopsy confirmed this presumptive diagnosis. The rarity of renal sinus AMLs extending into the pelvicalyceal system underscores the importance of recognizing this entity and distinguishing relatively benign PEComas from malignant urothelial neoplasms.

This case was presented at the 5th International RRF Conference, hosted by the Radiology Residents Forum on October 13, 2024, at King’s College London.

## Case presentation

A 23-year-old female was evaluated in the urology clinic for symptoms of right flank pain and hematuria. The initial workup included a non-contrast CT scan of the kidneys, ureters, and bladder. The imaging revealed that the right kidney was enlarged but maintained smooth contours.

**Figure 1:**
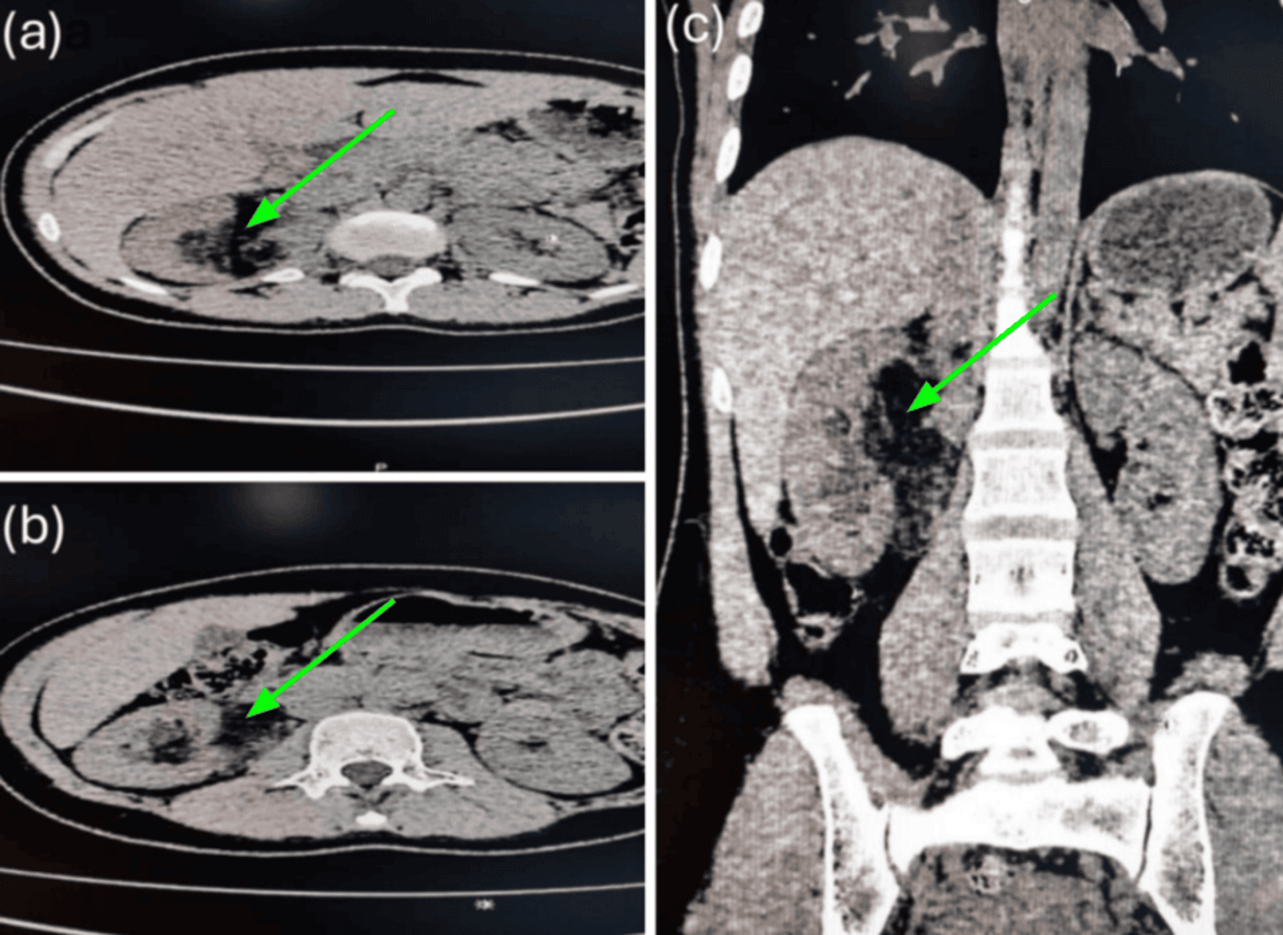
(A) CT KUB axial image showing a large, irregular, ill-defined, heterogeneous, predominantly fat-attenuating lesion with focal areas of soft tissue attenuation involving the pelvicalyceal system of the right kidney. (B) CT KUB axial image demonstrating extension of the lesion into the dilated renal pelvis. (C) CT KUB coronal reformatted image showing an abrupt transition at the right pelvicalyceal junction, suggestive of ureteropelvic junction obstruction with mild to moderate dilatation of the right pelvicalyceal system. KUB, kidneys, ureters, and bladder

A significant finding was a large, irregular, poorly defined, heterogeneous mass located within the pelvicalyceal system of the right kidney. The lesion was predominantly composed of fat-attenuating tissue with interspersed areas of soft tissue attenuation. It appeared to arise from the superior pole and extended into the dilated renal pelvis. Additionally, imaging demonstrated an abrupt transition point at the right ureteropelvic junction, indicative of obstruction. This resulted in mild to moderate upstream dilatation of the right pelvicalyceal system (Figure 1).

The radiological features supported a presumptive diagnosis of an AML originating in the renal sinus with intrapelvicalyceal extension. This diagnosis was subsequently confirmed by histopathological analysis of the excisional biopsy specimen.

## Discussion

AMLs are the most prevalent benign renal tumors, composed of vessels, smooth muscle, and fat. They usually arise in the renal cortex and are often discovered incidentally in female patients in their mid-50s. AMLs located in the renal sinus with extension into the pelvicalyceal system represent an uncommon presentation [9]. Kamath et al. (2010) reported five cases of renal sinus AMLs, describing MRI features such as well-circumscribed margins, insinuation around the collecting system, and localized hydrocalicosis without generalized hydronephrosis. Their findings suggested that conservative management is appropriate when diagnostic imaging reliably characterizes AMLs [10]. The present case is consistent with these imaging features, with the added findings of mild-to-moderate hydronephrosis and ureteropelvic junction obstruction, highlighting the obstructive potential of these lesions.

Metro et al. (2000) demonstrated that percutaneous biopsy can accurately diagnose renal sinus AML and preserve renal function [11]. This contrasts with earlier reports in which misdiagnosis led to unnecessary nephrectomies. In the current case, biopsy confirmation enabled a targeted excision of the lesion, reinforcing the importance of histopathological evaluation when imaging alone is inconclusive or nonspecific.

While classic AMLs larger than 4 cm are typically associated with bleeding risk, renal sinus AMLs are more likely to present with obstruction. Zagade et al. (2022) described a case of urinary obstruction due to AML that was successfully treated with embolization, underscoring the availability of multiple treatment options [12]. In our case, no evidence of hemorrhage or vascular invasion was noted, and surgical excision was deemed appropriate.

## Conclusions

This case demonstrates the characteristic imaging findings of a renal sinus AML with pelvicalyceal extension but is unusual due to its obstructive presentation in a younger patient. Percutaneous biopsy facilitated nephron-sparing surgery, consistent with prior recommendations, and helped the patient avoid unnecessary radical procedures. This case also emphasizes the complementary roles of imaging and histopathology in guiding conservative management of these rare presentations.
